# Sciatica

**Published:** 1875-02

**Authors:** 


### SCIATICA.

Is a pain more or less agonizing, beginning at the hip and running along the course of the sciatic nerve? It is really neuralgia in the hip—a nerve-ache, as tooth-ache is neuralgia of the tooth. Every ache or pain a man has is "neuralgia," having different names, according to the locality, as face-ache, head-ache, and the like.

As all aches arise from too much blood in the part, whatever diminishes the amount of blood there, either by abstracting it, as by leeches, irritation or otherwise, relieves the pain; or it may be relieved by attracting the excess of blood to some other part of the body, as by entering a dentist's office a tooth-ache often disappears, because a feeling of apprehension of suffering arises. This is in the brain, and the blood is drawn there in quantities proportioned to the apprehension. Many a person has taken a seat at the dinner table feeling badly, or having a head-ache or some other pain, but which disappeared during the meal, because the food excited the stomach, and thus attracted a surplus of blood there to the relief of the ailing part. It is on this principle that violent sciatica has been relieved at once, and finally disappeared, by eating every two hours. A man followed this plan for six months before the sciatica wholly disappeared, and about that time a permanent inflammation of the stomach had been engendered, which was simply the pain, the excess of blood, the congestion transferred to another spot, but far more dangerous, because inflammation of the stomach sometimes kills in a few days. Many a mother has given soothing syrup for the Summer complaint in her infant, too much blood on the inner surface of the bowels, or other pain. The symptoms cease, but the soothing syrup, like any other preparation of opium, affects the brain and draws there an excess of blood, and in a day or two, or less, the child has convulsions, epilepsy, or water on the brain, and dies, after having been "cured" by soothing syrup ! !

Hence the folly of allowing the administration of any medicine, however simple it is asserted to be, unless under the supervision of an intelligent and experienced physician. The lesson is, that no one ought to feel certain of being "cured" of anything until it has been ascertained that it has not changed its place or assumed another guise.

